# Blinded rechecking of sputum smear microscopy performance in public health facilities in Tigray region, Northern Ethiopia: Retrospective cross sectional study

**DOI:** 10.1371/journal.pone.0239342

**Published:** 2020-10-07

**Authors:** Letebrhan Weldemhret, Ataklti Hailu, Goyitom Gebremedhn, Hadish Bekuretsion, Gebreselassie Alemseged, Gebremicheal Gebreegziabher, Gebrhiwot Tesfahuney, Tesfaye Berhe, Lemlem Legesse, Kelali Kalayu, Mesfin Tesfay, Kibrti Mehari

**Affiliations:** 1 Laboratory Research Diagnostic and Quality Assurance Directorate, Tigray Health Research Institute, Mekelle, Tigray, Ethiopia; 2 Challenge TB, Mekelle, Tigray, Ethiopia; The University of Georgia, UNITED STATES

## Abstract

**Introduction:**

Tuberculosis disease is the leading cause of death worldwide along with HIV/AIDS. Sputum smear microscopy plays an essential role for initial TB diagnosis and treatment follow up. But, misdiagnosis of sputum smear microscopy revealed a high economical crisis and missing of active TB cases. This study was aimed to determine blinded rechecking of sputum smear microscopy performance in public health facilities in Tigray region, Northern Ethiopia.

**Materials and methods:**

A cross sectional retrospective study was conducted from January, 2017 to December, 2018 year. Data was collected retrospectively using electronic and paper based in Tigray health research institute. The data was analyzed using the SPSS version 25 software. The sensitivity, specificity, positive predictive value, and negative predictive value of the smear readings were calculated using 2X2 contingency table. The reading agreement between the microscopic center and reference center was determined using kappa statistics.

**Results:**

A total of 23,456 blinded rechecked smear results were reviewed. In average, the performances of sputum smear quality were 61%, 68%, 64%, 66%, 62% and 75% for specimen quality, staining quality, smear size, smear thickness, smear evenness and smear cleanliness respectively. Of the total error (0.48%) reported, 0.25%, 0.19% and 0.085% were false positive, false negative and quantification errors respectively. The concordance rate of health facilities for smear reading was increased to 90% by the end of 2018. Overall, the sensitivity, specificity, PPV, and NPV of the smear readings were 95%, 99.7%, 93% and 99.8% respectively. Likewise, the smear reading agreement was also perfect with kappa value, 0.87.

**Conclusion:**

The overall performance of public health facilities for blinded rechecking of smear microscopy was satisfactory. But, the high false positive and false negative errors found calls for continuous evaluation and monitoring of the health facilities by reference center.

## Introduction

Tuberculosis (TB) disease is caused by *Mycobacterium tuberculosis* (MTB) invariably affects the lungs [1]. It may also disseminate to other parts of the body via the blood stream, the lymphatic system, via the airways or by direct extension to other organs [1]. Moreover, MTB is an intracellular human pathogen and it may establish lifelong infection [2].

Tuberculosis is one of the top ten causes of death worldwide ranking above HIV/AIDS which is caused by single infectious agent [3, 4]. It was estimated that10 million people fall ill with TB each year [3]. Sputum smear microscopy plays indispensable role for initial TB diagnosis and treatment progression [5]. This method is widely applicable and identifies active tuberculosis which limits and controls active TB transmission [2, 6, 7]. Moreover, TB diagnostic laboratory and effective treatment program are essential for effective tuberculosis control in all over the world in resource scarce areas [5, 8]. But, unreliable smear microscopy result put the patient to high economical crisis and continuous transmission of the infection to the community [3, 5]. As a result quality assured smear microscopy TB detection is a great concern for TB control program [5].

Moreover, TB detection and treatment monitoring program will meet the national tuberculosis program through extensive implementation of external quality assessment (EQA) system and continuous monitoring of sputum smear microscopy [5, 8]. External quality assessment consists of proficiency panel testing, onsite evaluation and random blinded rechecking (RBRC) of smear slides [8, 9]. From these, fully functional RBRC is implementable and potentially measures the overall laboratory performance [8]. The most common errors encountered with smear microscopy were in appropriate specimen quality, in appropriate smearing technique, poor staining, bad microscopy and misreading or identification acid fast bacilli [5, 8, 9]. To maintain and meet the national TB control program, quality assured sputum smear microscopy is essential [9]. Furthermore, world health organization (WHO) has recommended evaluating the performance of TB microscopy centers after successful implementation of RBRC [8].

Few years back RBRC of sputum smear microscopy was decentralized to selected EQA centers throughout the country. But, the performance and effectiveness of the program was not known clearly since it was decentralized in the study region. In addition, once the RBRC was decentralized, the performance of the microscopic center should be evaluated for the effectiveness and continuality [8, 9]. As per us our knowledge, there was no previous research work regarding this in the study region. So, this study was aimed to assess blinded rechecking of sputum smear microscopy performance in public health facilities in Tigray region, Northern Ethiopia.

## Materials and methods

### Study setting

This study was conducted in Tigray region which is located in the Northern part of Ethiopia. The region has seven administrative zones, one special zone, 52 districts and 799 Kebeles. Based on the 2007 census projection, Tigray has an estimated total population of 4.8 million people over an area of 50,078.64 square kilometers. Majority (80.5%) of the population live in rural areas, while 19.5% are urban dwellers [10]. The region has 257 public health facilities includes 2 specialized referral hospitals, 15 general hospitals, 22 primary hospitals, 218 health centers and one research institute. Random blinded rechecking of sputum smear microscopy was decentralized to selected EQA centers throughout the region. All the TB microscopic centers once enrolled in the program, they are expected to participate quarterly in the program. There are also selected EQA center which are responsible for the blinded rechecking of the smears. This study was conducted in public health facilities which were enrolled in RBRC program for AFB smear microscopy.

### Study design and period

A cross sectional retrospective study was conducted from January 1^st^, 2017 to December 30, 2018 in Tigray region, Northern, Ethiopia.

### Study population

All public health facilities which were enrolled and participated in RBRC of sputum smear microscopy during the study period.

### Exclusion criteria

Public health facilities which were enrolled but were not actively participated in RBRC of sputum smear microscopy during the study period.

### Sample size and sampling technique

The sample size of this study was included all public health facilities which were enrolled and actively participated in RBRC of smear microscopy. So, the sample size of this study was included all public microscopic centers which were participated in blinded rechecking of sputum smear microscopy during the study period. Our electronic data base indicated that 200 public health facilities with a minimum two times a year were participated in blinded rechecking. Hence, 200 public health facilities were included in this study.

### Data collection tools

The data were collected retrospectively using electronic and paper based data collection tools in Tigray health research institute. Smear slides for random blinded rechecking was collected using lot quality assurance system which is a valid statistical sampling. In all woreda, there is a trained TB focal person who selects and transports the sputum smear slides to respected EQA center quarterly. The number of smear slides taken from each health facility depends on annual negative slide volume (ANSV), slide positivity rate (SPR), given sensitivity (80%), zero acceptance number and 95% confidence interval [9]. Once the smear slides arrived in the EQA center, the quality officer distributed to all laboratory personnel (first controller) blinded to the results of the peripheral microscopy. The results of the first controller were collected and compiled, if discordant happened the slide was given to the second controller and final result was generated from this. Discordances in microscopic centers were solved by travelling to microscopic center or inviting microscopic center personnel to EQA center. When the peripheral laboratory and the result of EQA center reached an agreement a corrective action was implemented by EQA center and monitored for the effectiveness of the action taken.

### Statistical data analysis

The data were analyzed using the Statistical Package for Social Sciences (SPSS) version 25 software. False negative and false positive errors were calculated using standard definitions [8]. If the microscopic center indicates a negative result while the EQA center reported positive, it is considered as false negative (FN). Positive result by the microscopic center while the EQA center reported negative result is categorized as false positive (FP). The sensitivity, specificity, positive predictive value (PPV), and negative predictive values (NPV) of the smear readings were calculated using the EQA center controller’s final result as a gold standard, per the national guideline [9]. Similarly, the reading agreement between the microscopic center and EQA center (controller) was determined using kappa statistics.

### Definition of terms

High False Negative (HFN);—is a 1+ to 3+ positive smear that is misread as negative.

High False Positive (HFP);—is a negative smear that is misread as 1+ to 3+ positive.

Low False Negative (LFN);—is a scanty (1–9 AFB /100 fields) positive smear that is misread as negative.

Low False Positive (LFP);—is a negative smear that is misread as a scanty (1–9 AFB / 100 fields) positive.

Quantification error (QE);—is the error happened when the difference grading report of a positive smear reading is greater than one compared to the stated quantification.

Major error:- is the most critical error which has the highest potential impact on patient management, and can result in an incorrect diagnosis or improper management of a patient [9].

Minor error:—is the error that may have lowest impact patient management. This type of error is considered less serious in evaluating laboratory performance.

### Ethical consideration

Ethical clearance was obtained from Tigray health research institution, institutional review board (THRI-IRB). Official written letter was also obtained from Tigray health research institute. This study has not direct contact with patient or patient sample, since all the data used were available in Tigray health research institution data base from routine program activities. Likewise, the IRB was waived the requirement for informed consent. This all were included and provided in the letter obtained from THRI-IRB.

## Results

### Health facilities trend of participation in blinded rechecking smear microscopy

In this study a total of 200 health facilities were participated from January, 2017 to December, 2018 year. The participations of health facilities for RBRC program were increased throughout the two consecutive years. In 2017 year in quarter (Q) four, the participations of health facilities were improved by 10% from quarter one. But, by the end of the 2018 year, the participations were decreased.

### Smear quality assessment from January, 2017 to December, 2018 year in public health facilities

A total of 23, 456 smear results were reviewed from 2017 to 2018 year. The specimen quality in quarter (Q) one of 2017 year was 40%. Likewise, the performance of specimen quality was 71% in 2018 year. In 2017 year, the performances of staining quality were 58%, 56%, 64% and 68% in Q1, Q2, Q3 and Q4 respectively. In 2017 year, the performances smear quality indicators were 53%, 61%, 55%, 59%, 54% and 70% for specimen quality, staining quality, smear size, smear thickness, smear evenness and smear cleanliness respectively. Moreover, in 2018 the specimen quality, staining quality, smear size, smear thickness, smear evenness and smear cleanliness were 69%, 74%, 72%, 72%, 70% and 80% respectively. In average, the performances of smear quality indicators for specimen quality, staining quality, smear size, smear thickness, smear evenness and smear cleanliness were 61%, 68%, 64%, 66%, 62% and 75% respectively (Table 1).

**Table 1:** Trends of participation and smear quality assessment in public health facilities from January, 2017 to December, 2018 year.

Year (GC)	Quarter (Q)	No. of participating HF	Sampled smear slides (N)	Specimen quality (%)	Staining quality (%)	Smear size (%)	Smear thickness (%)	Smear evenness (%)	Smear Cleanliness (%)
2017	Q1	118	3206	40	58	45	54	43	60
	Q2	128	2918	50	56	61	67	50	70
	Q3	138	3844	53	64	49	58	56	64
	Q4	125	3005	69	68	65	60	67	73
Average				53	61	55	59	54	70
2018	Q1	125	2960	69	69	73	68	70	71
	Q2	121	3057	70	72	70	70	72	77
	Q3	96	2531	67	80	71	74	69	87
	Q4	95	1935	71	76	77	76	70	85
Average				69	74	72	72	70	80
	Total		23,456	61	68	64	66	62	75

### Performance of health facilities for smear microscopy reading from January, 2017 to December, 2018

A total of 22,602 negative and 854 positive smear results were reviewed. Of these, 0.48% (122) was found discrepant smear reading error. These error consists of 0.25%, 0.19% and 0.085% for false positive (FP), false negative (FN) and quantification error (QE) respectively. In 2017 year, 0.2% of FP and 0.24% of FN discrepancies were found. Likewise, in 2018 year, a FP of 0.3% and 0.12% of FN discrepant findings were observed.

In 2017 year, of the reviewed smears, 0.4% (13), 0.45% (13), 0.78% (30) and 0.6% (18) errors were identified in Q1, Q2, Q3 and Q4 respectively. Likewise, in 2018 year, 0.64% (19), 0.43% (13), 0.49% (10) and 0.3% (6) errors were observed in Q1, Q2, Q3 and Q4 respectively (Table 2). The sensitivity, specificity, PPV, and NPV of the smear readings were 94.9%, 99.5%, 96% and 99.7% respectively in 2017 year. In addition, in 2018 year the sensitivity, specificity, PPV, and NPV of smear readings were also 97.2%, 99.6%, 94.5% and 99.8% respectively. The overall sensitivity, specificity, PPV, and NPV smear readings in two consecutive years were 95%, 99.7%, 93% and 99.8% respectively. Moreover, the smear reading agreement between the microscopic and reference center was also almost perfect agreement (Kappa value = 0.87).

**Table 2:** Number of reviewed smears and trend of false positive and negative errors from January, 2017 to December, 2018 year.

Year (GC)	Quarter	Total slide collected	Microscopic local result		EQA (reference) result		False positive result	False negative result	QE	Total
			**Pos**	**Neg**	**Pos**	**Neg**				
2017	Q1	3206	119	3087	116	3090	6	3	4	13
	Q2	2918	190	2728	191	2727	4	5	4	13
	Q3	3844	136	3708	140	3704	11	15	4	30
	Q4	3005	86	2919	89	2916	5	8	5	18
2018	Q1	2960	98	2862	85	2875	16	3	0	19
	Q2	3057	110	2946	107	2949	7	4	2	13
	Q3	2531	49	2482	48	2483	5	4	1	10
	Q4	1935	66	1869	64	1871	4	2	0	6

**Fig 1:**
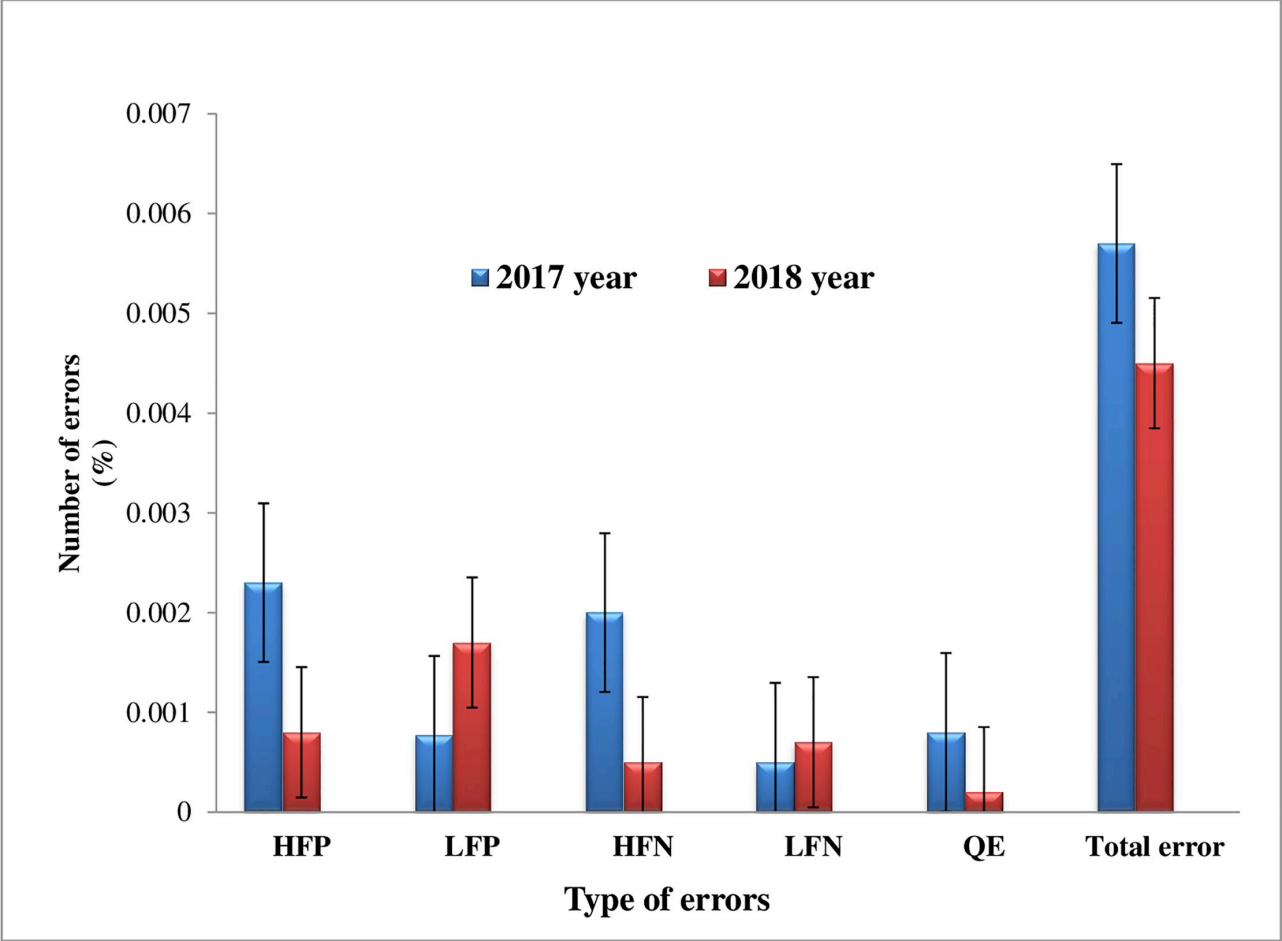
Trends of error for AFB smear microscopy reading (%). HFP: High false positive, LFP: low false positive, HFN: High false negative, LFN: low false negative, QE: Quantification error.

In 2017 year, the concordance rates of health facilities were 83% (165). Moreover, in 2018 year, the concordance rate of health facilities was 90% (180). The overall concordance rate of health facilities was 81% in two consecutive years. High false positive errors were reported by 21 health facilities. One or greater HFN error were reported by15 health facilities. The HFP and/or HFN errors were reported by 15% (30) of health facilities. Of the total reviewed smears in 2017 year, the total error or discrepancy smear reading was found 0.57%. Likewise, the total error in 2018 was 0.45% (**Fig 1**). The high false positive (HFP) and HFN errors were 0.23% and 0.2% respectively in 2017 year. In 2018, a HFP of 0.08% and HFN of 0.05% were identified. The quantification errors (QE) were 0.08% and 0.02% in two consecutive years. The low false positive (LFP) and low false negative (LFN) smear reading errors were 0.17% and 0.07% respectively in 2017 year (**Fig 1**).

## Discussion

Sputum smear microscopy has indispensable role for MTB diagnosis mainly in developing countries due to its cost effectiveness and easy implementation at all peripheral level. In this study we have tried to address the performance of RBRC of sputum smear microscopy and errors encountered with smear reading. The proportion of health facilities participation in blinded rechecking was increased throughout the study period. But, by the end of the 2018 year, the participation was decreased by 11% from the beginning of the study. The possible reason for this was routine sputum smear microscopy was replaced by Gene X pert method for MTB and rifampicin resistance detection in all primary and above public hospitals since that time.

In 2017 year, the performance of specimen quality at the beginning was 53% and reached to 69% by the end of 2018 year. This was in line with the study conducted in Addis Abeba indicated that the specimen quality was increased from 40% to 80% over a period of time [11]. Likewise, the staining quality, smear size, smear thickness, smear evenness and smear cleanliness were improved to 74%, 72%, 72%, 70% and 80% respectively by the end of December, 2018. This indicated that continuous participation of microscopic center in random blinded rechecking of smear microscopy potentially improves the quality of smears which increases the reliability and accuracy of AFB results. Similar studies suggested that a staining quality of 71% at base line and reached to 81% and a smear thickness from 62% to 70% after implementation of blinded rechecking smears [11, 12]. Other studies in Ghana and Philippines also showed similar findings [13, 14]. In contrast, the performances of smear quality indicators were higher than to study in Addis Abeba [15]. The difference was attributed to the training status of the laboratory personnel and practice of EQA for AFB smear microscopy.

In average, a total error or discrepancy smear reading of 0.48% was found over two years. This finding was in line with the study conducted in Addis Abeba [15]. But, it was lower than to research findings in India (1.7%) and democratic republic of Congo (10.4%) [16, 17]. The difference might be due to the difference in study period and sample size included in the study. The total discrepant error investigated in Q1 (0.4%), Q2 (0.45%), Q3 (0.78%) and Q4 (0.65) of 2017 year were increased throughout the year. During initial implementation of the random blinded rechecking program, it is obvious that the number of smear reading errors could be increased as a result of no previous system to handle such discrepancy. But, in 2018 year, smear reading errors were decreased throughout Q1 (0.64%), Q2 (0.43%), Q3 (0.49) and Q4 (0.3%). Overall, the total smear reading error was decreased to 0.45% by the end of 2018 year.

In this study the FN errors were decreased from 0.24% in 2017 to 0.12% in 2018 year. These findings were comparable with studies indicated that the FN errors were decreased from 7.6% to 1.6% over a period of time in Oromia and Amhara region [11]. In average, the FN (0.19) results of this study were comparable with the studies in Addis Abeba and Tajikistan [12, 18]. But, lower than the findings (3.5%) found in Eastern part of Ethiopia [19]. The possible reason for FN result is poor staining quality and lack of internal quality control practice [20, 21]. Likewise, it may also due to bad microscopy, inexperienced microscopist or inadequate training [7, 6].

The overall FP result of this study was 0.25% which was comparable with the study finding in Addis Abeba [12]. In contrast, the finding were incomparable with the study results in Ethiopia and India [15, 16]. The trend of FP error was steadily increased from 0.2% in 2017 to 0.3% in 2018 year. But, majorities of the FP errors were attributed to LFP error (0.17%) which is less serious to measure the laboratory performance due to the inherent problem of the smear microscopy in detecting or quantifying low amount of bacilli load. False positive errors are frequently happened due to insufficient decolorization, reagent precipitation and inexperienced microscopist [8, 9].

The overall sensitivity, specificity, positive predictive value, and negative predictive value of blinded rechecking smear microscopy was 95%, 99.7%, 93% and 99.8% respectively. These findings were in line with the study conducted in Oromia region [11]. Another studies were also indicated similar findings [12, 15]. Based on WHO guideline for external quality assessment of TB smear microscopy, a sensitivity of smear reading close to 95% is acceptable which supports our findings [8]. Likewise, the national guideline for external quality assessment of AFB smear microscopy indicated a sensitivity of 80% and greater is the optimal performance for blinded rechecking smear reading [9]. So, overall performances of health facilities for blinded rechecking smear reading were within the acceptable cut of value [8, 9]. The smear reading agreement between the microscopic and reference center was also almost perfect agreement with kappa value = 0.87. Similar study reported in East Harerge [19]. But, the reading agreement was incomparable to other study findings [12, 15, 22]. The difference might be due to variation in sampling volume and EQA evaluation and monitoring system. Because including large volume of samples in blinded rechecking program was revealed a large performance acceptance [8].

Majority of the health facilities (81%) have showed concordance smear reading with the reference center. Moreover, the concordance rate of health facilities for smear reading was increased from 82.5% in 2017 year in to 90% in 2018 year. Researchers in Oromia and Amhara region suggested that microscopic center with no smear reading errors were improved from 77.9% at the beginning of the study to 90.5% [11]. In this study 21 and 15 health facilities were reported HFP and HFN results respectively. In addition, at least one major error was reported by 15% of health facilities. Any major error with smear reading is a serious error which indicating misclassification of the diseases and patient management. Based on international EQA guideline for TB smear microscopy error interpretation any HFP, two or greater HFN findings and >3 LFN reported by microscopic center were potential source of error for unacceptable performance [8]. Hence, the HFP and HFN errors reported were indicated unacceptable performance. High false positive errors frequently happened due to poorly maintained microscopy, poor staining, reagent precipitation and lack of adherence to internal quality control [5, 8]. But, higher numbers of HFP were associated with inexperienced microscopist, bad microscopy or administrative errors during record keeping [9].

## Conclusions

Overall, public health facilities participated in random blinded rechecking of sputum smear microscopy of had shown an acceptable performance with good smear reading agreement. The quality of sputum smears were improved throughout the quarter’s participation of the health facilities in RBRC program. Moreover, the concordance rates of health facilities for smear reading were becoming improved. This is an implication of the program for improving the quality smear microscopy to achieve the country national policy for successful end TB strategy. But, the high false positive and high false negative errors found within the microscopic center were above the cut of value set in international EQA guideline for blinded rechecking sputum smear microscopy. Hence, all EQA centers have the responsibility to monitored for any identified major errors with smear readings.
